# Echocardiographic characteristics of PRKAG2 syndrome: a research using three-dimensional speckle tracking echocardiography compared with sarcomeric hypertrophic cardiomyopathy

**DOI:** 10.1186/s12947-022-00284-3

**Published:** 2022-05-05

**Authors:** Lu Tang, Xuejie Li, Nianwei Zhou, Yingying Jiang, Cuizhen Pan, Xianhong Shu

**Affiliations:** Department of Echocardiography, Zhongshan Hospital, Shanghai Institute of Cardiovascular Diseases, Shanghai Institute of Medical Imaging, Fudan University, Shanghai, China

**Keywords:** PRKAG2 syndrome, Hypertrophic cardiomyopathy, 3D STE, Strain, GLS

## Abstract

**Background:**

PRKAG2 syndrome is a rare disease characterized as left ventricular hypertrophy (LVH), ventricular preexcitation syndrome, and sudden cardiac death. Its natural course, treatment, and prognosis were significantly different from sarcomeric hypertrophic cardiomyopathy (HCM). However, it is often clinically misdiagnosed as sarcomeric HCM. PRKAG2 patients tend to experience delayed treatment. The delay may lead to adverse outcomes. This study aimed to identify the echocardiographic parameters which can differentiate PRKAG2 syndrome from sarcomeric HCM.

**Methods:**

Nine PRKAG2 patients with LVH, 41 HCM patients with sarcomere gene mutations, and 202 healthy volunteers were enrolled. Clinical characteristics, conventional echocardiography, and three-dimensional images were recorded, and reviewed by an attending cardiologist. We evaluated the parameters of left ventricular strains from three-dimensional speckle tracking echocardiography (3D STE) by TomTec software. Receiver operating characteristic (ROC) curves analysis was used to assess clinical and echocardiographic parameters’ differential diagnosis potential.

**Results:**

The heart rate (HR) of the PRKAG2 group was significantly lower than both the healthy group (53.11 ± 10.14 vs. 69.22 ± 10.48 bpm, *P* < 0.001) and the sarcomeric HCM group (53.11 ± 10.14 vs. 67.23 ± 10.32 bpm, *P* = 0.001). The PRKAG2 group had similar interventricular septal thickness (IVS), posterior wall thickness (PWT), and maximum wall thickness (MWT) to the HCM group (*P* > 0.05). The absolute value of GLS in the PRKAG2 group was significantly higher than HCM patients (-18.92 ± 4.98 vs. -13.43 ± 4.30%, *P* = 0.004). SV calculated from EDV and ESV in PRKAG2 syndrome showed a higher value than sarcomeric HCM (61.83 ± 13.52 vs. 44.96 ± 17.53%, *P* = 0.020). The area under the ROC curve (AUC) for HR + GLS was 0.911 (0.803 -1). For HR + GLS, the sensitivity and specificity of the best cut-off value (0.114) were 69.0% and 100%, respectively.

**Conclusions:**

PRKAG2 patients present deteriorated LV diastolic function and preserved LV systolic function. Bradycardia and preserved GLS are useful to identify PRKAG2 syndrome from sarcomeric HCM, which may be beneficial for clinical decision-making.

**Supplementary Information:**

The online version contains supplementary material available at 10.1186/s12947-022-00284-3.

## Background

PRKAG2 syndrome is a rare cardiac abnormality manifesting left ventricular hypertrophy (LVH) with electrophysiological abnormalities caused by mutations in the gene of the γ2 subunit (*PRKAG2*) of adenosine monophosphate-activated protein kinase (AMPK) [1, 2]. AMPK facilitates cellular glucose and fatty acid metabolic pathways. It acts as an enzymatic modulator of adenosine triphosphate utilizing pathways by phosphorylation [3]. The defect of the *PRKAG2* gene alters AMPK’s activity resulting in excessive cellular glycogen storage. The perturbation in the exquisite regulation of cardiac metabolism leads to LVH, ventricular preexcitation, supraventricular arrhythmias, implantation of a pacemaker, and even sudden cardiac death [4–6]. Although PRKAG2 syndrome’s pathophysiology distinguishes it from hypertrophic cardiomyopathy (HCM), the phenotype of LVH can mimic sarcomeric HCM and interfere with earlier differential diagnosis [7]. We aimed to identify the clinical and echocardiographic parameters that could differentiate PRKAG2 syndrome from sarcomeric HCM.

## Methods

### Study design

This cross-sectional, retrospective study included 252 participants of three groups: nine PRKAG2 patients with LVH, 41 HCM patients with sarcomere gene mutations, and 202 healthy volunteers. We excluded patients who were diagnosed with hypertension, aortic stenosis, or coronary heart disease. Mutations in *PRKAG2* and sarcomere genes were identified by whole-exome sequencing and confirmed by Sanger sequencing. The Ethics Committee of Zhongshan Hospital Fudan University approved this study. Informed consent was obtained from the subjects before participation.

### Transthoracic echocardiography

Transthoracic echocardiography examinations were performed by an experienced licensed echocardiographic physician using Philips iE33 ultrasound machines (Philips Medical System, Andover, MA, USA). Standard apical four-chamber, apical two-chamber, apical long-axis, parasternal long-axis, and parasternal short-axis views were obtained with S5-1 transducer. Cardiac diameters were measured according to current guidelines [8]. Left ventricular wall asymmetry (LVWa) was the ratio of interventricular septum thickness (IVS) to left ventricular posterior wall thickness (PWT) measured in the parasternal long-axis view. Relative wall thickness (RWT) was measured as the ratio of 2* PWT over LV end-diastolic diameter (LVEDD). Left ventricular ejection fraction (LVEF) was calculated using the biplane Simpson’s method.

Early and late transmitral velocity (E, A) were obtained using pulsed-wave Doppler echocardiography at the mitral valve inflow. Also, early diastolic and systolic mitral annular displacement velocity (e’, s’) were acquired using tissue Doppler echocardiography.

**Fig. 1:**
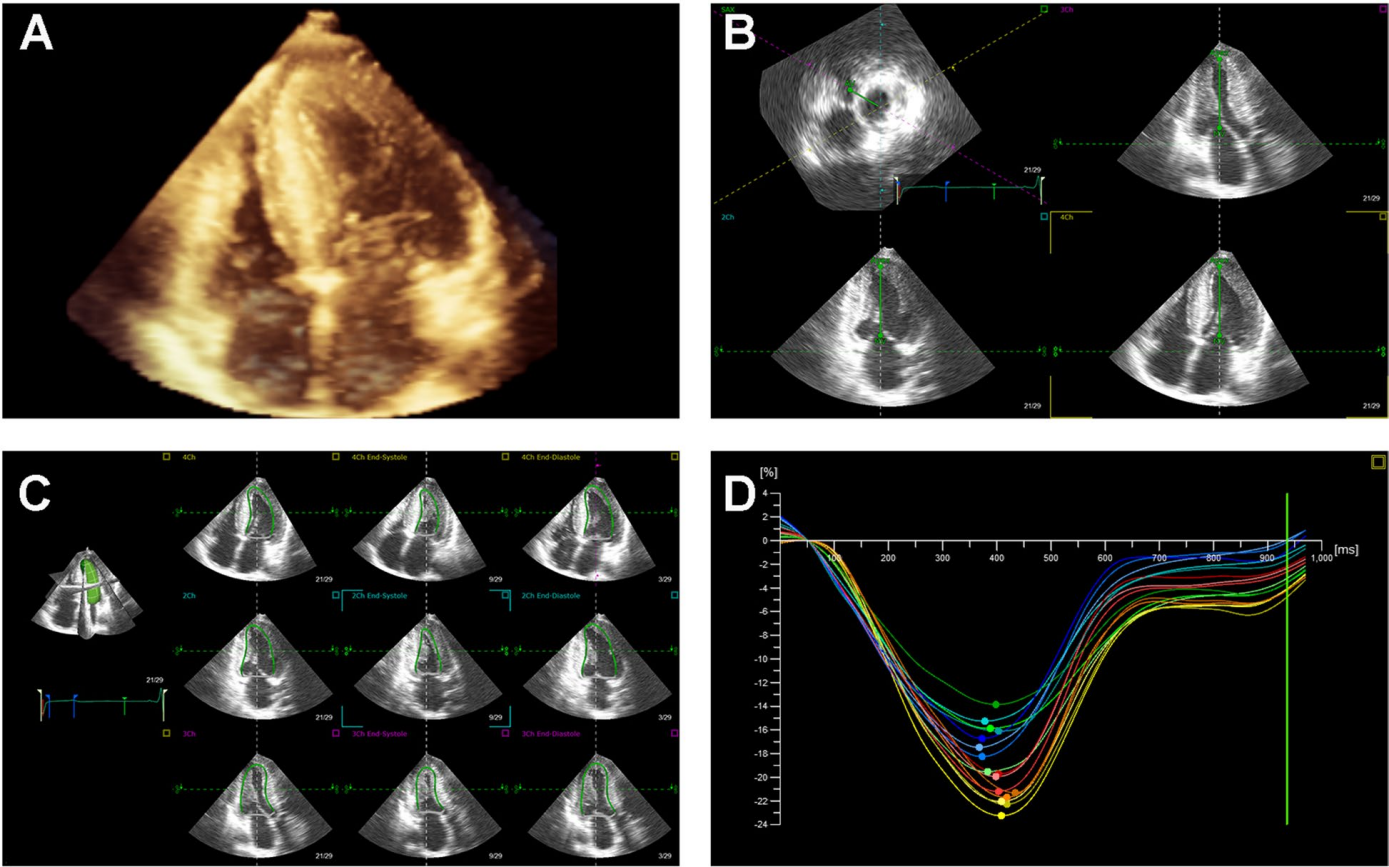
3D STE offline analysis. **A** An apical four-chamber 3D full volume image. **B** The points of the cardiac apex and mitral valve are manually adjusted for software automatic identification. **C** The endocardial border is traced and tracked in the apical triplane views. **D** An example of GLS obtained from 3D STE analysis

Three-dimensional images were recorded from the apical four-chamber view for four heartbeats using an X5-1 transducer. All 3D echocardiographic images were analyzed offline by TomTec software (TomTec Imaging Systems, Unterschleissheim, Germany). As shown in Fig. 1, the left ventricular (LV) endocardial border could be automatically tracked and then manually adjusted. Then, stroke volume (SV), ejection fraction (EF), global longitudinal strain (GLS), global circumferential strain (GCS), twist, torsion, systolic timing dispersion index (SDII), and systolic timing deviation index (SDI) of LV were calculated based on speckle tracking echocardiography (STE).

Echocardiography, electrocardiography, and blood pressure measurement were performed in all subjects at rest.

### Statistical analysis

Continuous variables were expressed as mean ± SD. Normality was determined by Kolmogorov–Smirnov normality test. T-test or Mann–Whitney U test was used to compare numerical variables between the PRKAG2 group and other groups. Fisher’s exact tests were used to analyze the differences between categorical variables. To evaluate the specificity and sensitivity of multiple variables to predict PRKAG2 syndrome, logistic regression was performed to combine parameters as a new predictor. Then, the receiver operating characteristic (ROC) curve was plotted to calculate the area under the curve (AUC) to distinguish the PRKAG2 group from the HCM group. Data was assessed by R software, version 3.6.0 (R Core Team, R Foundation for Statistical Computing). *P* < 0.05 was considered as statistically significant.

## Results

### Clinical characteristics and ECG findings

**Fig. 2:**
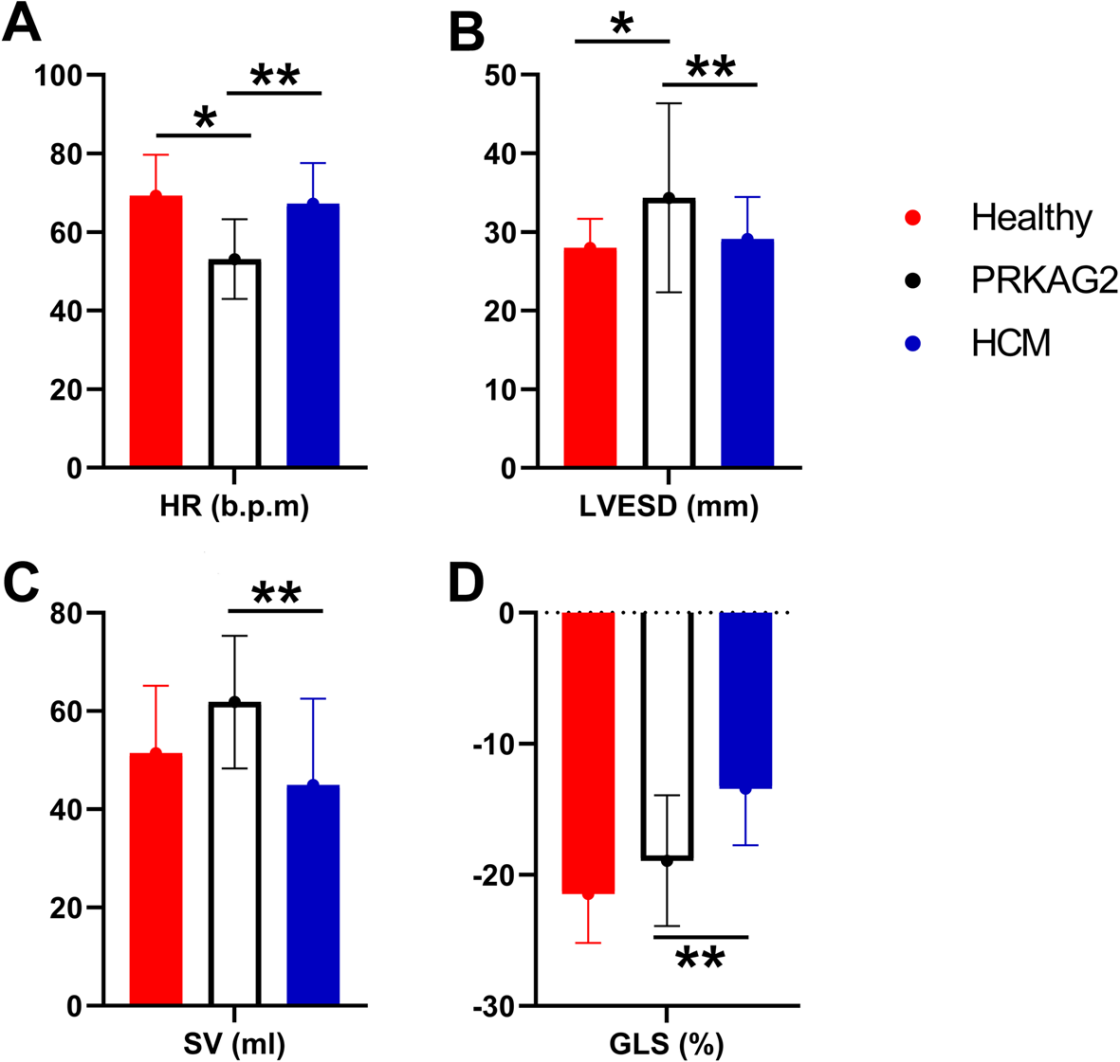
There were significant differences in HR, LVESD, SV, and GLS between PRKAG2 syndrome and sarcomeric HCM patients. HR and LVESD of PRKAG2 LVH also had significant differences compared with healthy volunteers. * refers to *P* < 0.05 between the PRKAG2 syndrome group and the healthy group. ** refers to *P* < 0.05 between the PRKAG2 syndrome group and the sarcomeric HCM group

Clinical characteristics and HR were presented in Table 1. In general, we observed nine patients with PRKAG2 syndrome in six independent families (Supplementary Fig. 1). One patient (IV-1 in Family D) suffered from intellectual disability and somnambulism for more than ten years. Two patients showed LV preexcitation. Six patients presented LVH on ECG images, and one patient demonstrated abnormal Q waves. The heart rate (HR) of the PRKAG2 group was significantly lower than both the healthy group (53.11 ± 10.14 vs. 69.22 ± 10.48, *P* < 0.001) and the HCM group (53.11 ± 10.14 vs. 67.23 ± 10.32 bpm, *P* = 0.001) (Fig. 2A). Four in nine PRKAG2 patients and 17 in 41 HCM patients received beta blocker treatment (*P* = 1.000).

**Table 1:** Clinical characteristics of the study population

Variable	PRKAG2	Healthy	HCM	P1	P2
Age, years	40.22 ± 14.01	42.04 ± 14.86	49.59 ± 12.75	0.719	0.056
Male/female	5/4	68/134	28/13	0.281	0.467
BSA, m^2^	1.74 ± 0.20	1.65 ± 0.19	1.78 ± 0.21	0.199	0.627
BMI, kg/m^2^	23.80 ± 2.77	23.39 ± 3.88	25.45 ± 3.54	0.771	0.256
HR, bpm	53.11 ± 10.14	69.22 ± 10.48	67.23 ± 10.32	< 0.001	0.001

P1: P values between the PRKAG2 group and the healthy volunteers, P2: P values between the PRKAG2 group and the sarcomeric HCM group. *BSA* Body surface area, *BMI* Body mass index, *HR* heart rate

### Parameters of conventional echocardiography

Conventional echocardiographic parameters were shown in Table 2. The PRKAG2 group had similar interventricular septal thickness (IVS), posterior wall thickness (PWT), maximum wall thickness (MWT), and left ventricular end-diastolic diameter (LVEDD) to the sarcomeric HCM group (*P* > 0.05). The LVWa of the PRAKG2 patients was significantly higher than that of the healthy volunteers (1.42 ± 0.52 vs. 1.05 ± 0.14 mm, *P* < 0.001), which was close to that of the HCM patients (1.42 ± 0.52 vs. 1.55 ± 0.63 mm, *P* = 0.551). RWT of the PRKAG2 group was significantly higher than that of the healthy group (0.48 ± 0.15 vs. 0.39 ± 0.07, *P* = 0.002). The left ventricular end-systolic diameter (LVESD) of the PRKAG2 group were significantly larger compared with the sarcomeric HCM group and the healthy volunteers (*P* < 0.05) (Fig. 2B). PRKAG2 syndrome patients demonstrated impaired LV diastolic function parameters, including A, e’ and E/e’. However, their LVEF remained in the normal range (PRKAG2 syndrome 62.67 ± 8.56 vs. healthy group 65.79 ± 6.88%, *P* = 0.189).

**Table 2:** Conventional echocardiographic parameters

Variable	PRKAG2	Healthy	HCM	P1	P2
AoD, mm	31.89 ± 2.71	30.39 ± 3.45	32.76 ± 3.09	0.200	0.441
LAD, mm	42.56 ± 7.40	33.65 ± 3.88	44.59 ± 7.70	< 0.001	0.475
IVS, mm	16.56 ± 6.31	8.84 ± 1.67	17.27 ± 5.51	< 0.001	0.733
PWT, mm	11.89 ± 3.76	8.48 ± 1.38	11.73 ± 3.36	< 0.001	0.585
LVWa	1.42 ± 0.52	1.05 ± 0.14	1.55 ± 0.63	< 0.001	0.551
MWT, mm	21.89 ± 6.45	9.05 ± 1.63	21.48 ± 5.97	< 0.001	0.853
LVEDD, mm	50.33 ± 8.38	43.64 ± 4.55	45.49 ± 5.38	0.005	0.107
LVESD, mm	34.44 ± 12.01	27.96 ± 3.71	29.12 ± 5.33	< 0.001	0.046
LVEF, %	62.67 ± 8.56	65.79 ± 6.88	64.22 ± 7.39	0.189	0.581
E, cm/s	76.84 ± 19.76	81.81 ± 17.46	72.23 ± 20.66	0.408	0.553
A, cm/s	51.67 ± 10.84	64.13 ± 16.98	66.03 ± 25.29	0.030	0.107
s’, cm/s	6.67 ± 1.91	10.01 ± 2.42	7.40 ± 1.38	< 0.001	0.186
e', cm/s	7.92 ± 2.38	12.04 ± 3.78	6.80 ± 1.96	0.001	0.208
E/e'	10.31 ± 4.10	7.34 ± 2.37	11.24 ± 4.45	< 0.001	0.578

P1: P values between the PRKAG2 group and the healthy volunteers, P2: P values between the PRKAG2 group and the sarcomeric HCM group. *AoD* aortic dimension, *LAD* left atrial dimension, *IVS* interventricular septum thickness, *PWT* posterior wall thickness, *LVWa* left ventricular wall asymmetry, *MWT* maximum wall thickness, *LVEDD* left ventricular end-diastolic diameter, *LVESD* left ventricular end-systolic diameter, *LVEF* left ventricular ejection fraction

### Parameters of speckle tracking imaging

The results of the 3D STE analysis were shown in Table 3. EDV and ESV from 3D STE also enlarged in PRKAG2 syndrome (*P* < 0.05). The differences were not significant compared with the sarcomeric HCM group (*P* > 0.05). However, SV calculated from EDV and ESV in PRKAG2 syndrome was larger than that of sarcomeric HCM (61.83 ± 13.52 vs. 44.96 ± 17.53%, *P* = 0.020) (Fig. 2C). The value of GLS in PRKAG2 syndrome was significantly better than that of HCM (-18.92 ± 4.98 vs. -13.43 ± 4.30%, *P* = 0.004) and near the healthy level (-18.92 ± 4.98 vs. -21.45 ± 3.73%, *P* = 0.083) (Fig. 2D). In terms of dyssynchrony, SDI increased in the PRKAG2 group compared with the control group (5.83 ± 1.17 vs. 4.59 ± 1.56%, *P* = 0.033). However, there were no significant differences of SDI or SDII between the PRKAG2 group and the HCM group.

**Table 3:** 3D STE parameters

Variable	PRKAG2	Healthy	HCM	P1	P2
EDV, ml	107.89 ± 34.79	81.16 ± 21.80	89.58 ± 32.80	0.002	0.183
ESV, ml	46.06 ± 25.80	29.72 ± 12.28	44.61 ± 21.57	0.001	0.874
SV, ml	61.83 ± 13.52	51.43 ± 13.73	44.96 ± 17.53	0.050	0.020
EF, %	59.78 ± 11.69	63.97 ± 8.51	50.97 ± 11.88	0.207	0.076
GLS, %	-18.92 ± 4.98	-21.45 ± 3.73	-13.43 ± 4.30	0.083	0.004
GCS, %	-29.27 ± 9.06	-31.17 ± 6.93	-23.99 ± 7.30	0.481	0.095
SDI, %	5.83 ± 1.17	4.59 ± 1.56	8.66 ± 5.12	0.033	0.098
SDII, %	11.70 ± 2.36	8.72 ± 5.85	12.27 ± 4.78	0.180	0.761
Twist, °	12.67 ± 3.11	13.88 ± 8.39	14.25 ± 8.78	0.704	0.643
Torsion, °/cm	1.55 ± 0.44	1.89 ± 1.14	1.85 ± 1.12	0.433	0.491

P1: P values between the PRKAG2 group and the healthy volunteers; P2: P values between the PRKAG2 group and the sarcomeric HCM group. *EDV* end-diastolic volume, *ESV* end-systolic volume, *SV* stroke volume, *EF* ejection fraction, *GLS* global longitudinal strain, *GCS* global circumferential strain, *SDI* systolic timing deviation index, *SDII* systolic timing dispersion index

### Parameters for differential diagnosis

**Fig. 3:**
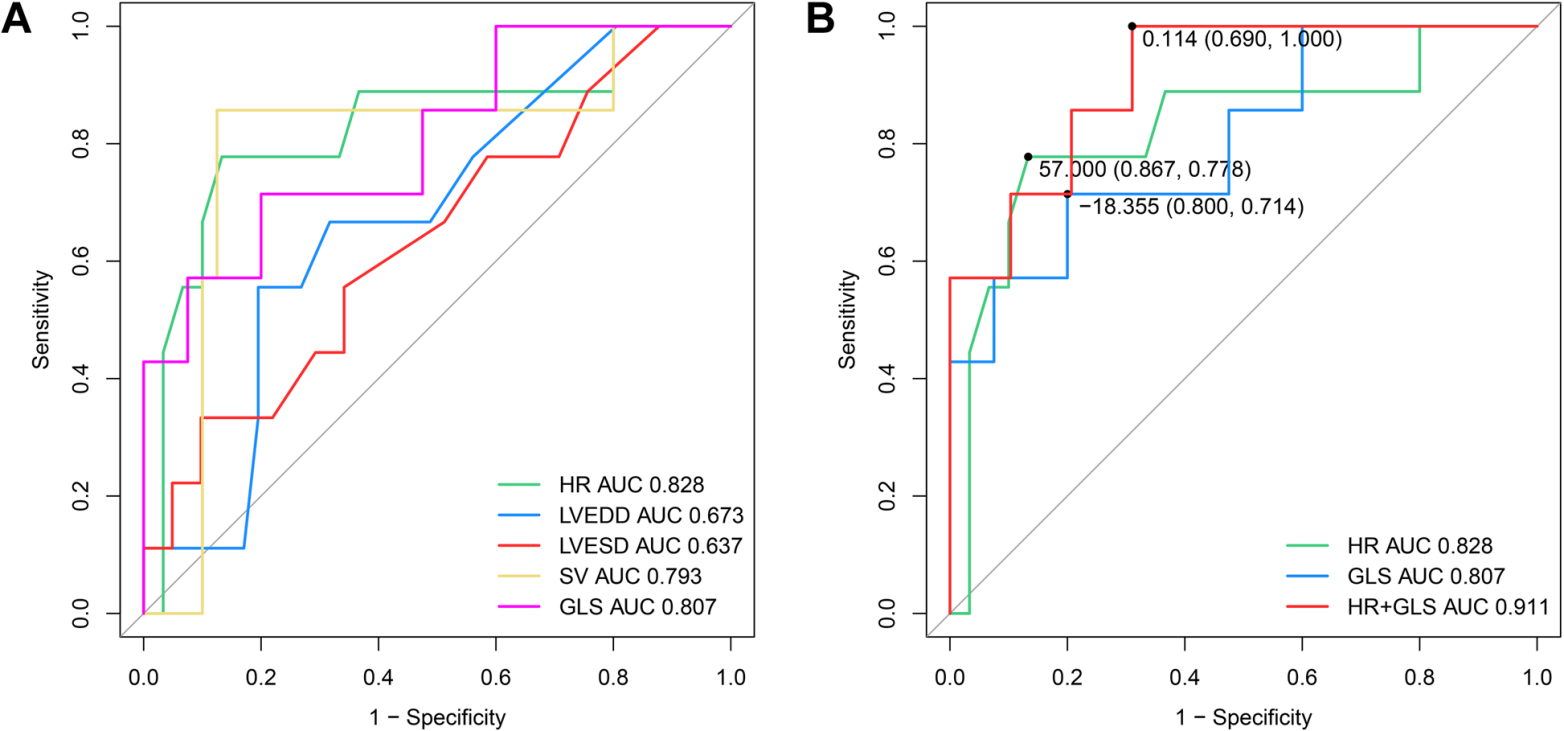
Comparison of ROC curve analysis for prediction of PRKAG2 syndrome in LVH patients. **A** The ROC curves are based on statistically significant parameters, including HR, LVEDD, LVESD, SV, and GLS. **B** The two parameters (HR and GLS) with the highest AUC were selected for Logistic regression analysis. We put the new predictor (HR + GLS) into ROC curve analysis and got the highest AUC of 0.911. The best cut-off value (0.114) sensitivity and specificity were 69.0% and 100%, respectively

The area under the curve (AUC) and the confidence interval of AUC from ROC analysis were demonstrated in Table 4 and Fig. 3A. Among them, the AUC of HR and GLS were the highest with the lower confidence limit > 0.6. We put HR and GLS into the Logistic regression test and got a new predictor of HR + GLS. The AUC for HR + GLS was 0.911 (0.803—1), which was higher than that of HR (0.828, 0.646—1) and GLS (0.807, 0.614—1) (Fig. 3B). For HR + GLS, the sensitivity and specificity of the best cut-off value (0.114) were 69.0% and 100%, respectively.

**Table 4:** ROC curve analysis results

Variable	AUC	95% CI	Cut-off value	Sensitivity	Specificity
HR	0.828	0.646–1	57	0.867	0.778
LVEDD	0.673	0.487–0.86	50.5	0.805	0.556
LVESD	0.637	0.427–0.846	35.5	0.902	0.333
SV	0.793	0.581–1	58.79	0.875	0.857
GLS	0.807	0.614–1	-18.355	0.800	0.714
HR + GLS	0.911	0.803–1	0.114	0.690	1.000

P1: P values between the PRKAG2 group and the healthy volunteers; P2: P values between the PRKAG2 group and the sarcomeric HCM group. *HR* heart rate, *LVEDD* left ventricular end-diastolic diameter, *LVESD* left ventricular end-systolic diameter, *SV* stroke volume, *GLS* global longitudinal strain

## Discussion

PRKAG2 syndrome is a progressive glycogen storage disease. It had been considered a part of HCM for several decades until 2001 when autosomal dominant inherited mutations in the *PRKAG2* gene were first observed to be responsible for this syndrome [6]. *PRKAG2* gene encodes the γ2 regulatory subunit of AMPK, enhancing glucose and lipids metabolism [9]. This rare disease is characterized by LVH, ventricular preexcitation, diverse arrhythmia, advanced heart failure, and sudden cardiac death. Almost 10% of PRKAG2 cases suffered from sudden death; more than 30% required pacemaker implantation [10]. Although PRKAG2 syndrome comprises less than one percent of unexplained LVH, the differential diagnosis is crucial because of its distinct natural history, poor prognosis, and active treatment strategies.

Echocardiography is a noninvasive, widely available, and first-line technique to identify and quantify LVH [11]. This study collected the parameters of conventional two-dimensional echocardiography, Doppler ultrasound, and 3D STE. We aimed to compare PRKA2G syndrome with healthy volunteers and sarcomeric HCM.

In literature, MWT of PRAKG2 syndrome is from 20 to 21 mm, which is similar to our measurements of 21.89 ± 6.45 mm [10, 12]. The RWT of PRKAG2 syndrome in this study was 0.48 ± 0.15, which reached the definition of concentric hypertrophy pattern (RWT > 0.42) [13]. These findings confirmed the concentric LVH pattern of PRKAG2 syndrome. However, we also observed asymmetric septal hypertrophy in our PRKAG2 patients (LVWa = 1.42 ± 0.52). This may reflect the diversity of PRKAG2 syndrome. Larger size studies are needed to find the exact ratio of hypertrophy patterns.

We evaluated LV diastolic function using parameters of Doppler echocardiography parameters (E, A, e’, E/e’). Most parameters indicated PRKAG2 LV diastolic dysfunction similar to sarcomeric HCM.

General HCM patients had worse GLS compared with healthy individuals [14]. Recently, STE has been reported to be a non-Doppler-oriented and angle-independent technique that helped detect different causes of LVH [15–17]. However, few articles focused on the STE parameters in PRKAG2 syndrome because of the low prevalence.

Interestingly, we observed preserved GLS and GCS in our PRKAG2 patients compared with healthy volunteers (GLS -18.92 ± 4.98 vs. -21.45 ± 3.73%, *P* = 0.083; GCS -29.27 ± 9.06 vs. -31.17 ± 6.93%, *P* = 0.481). However, in a recent study using STE, the GLS and GCS strains of PRKAG2 syndrome decreased as -13 ± 4.8% and -16.1 ± 4.4%, respectively [12]. Healthy controls were not involved in that study. Different echocardiographic systems, software, operators, and pathophysiological heterogeneity might contribute to the discrepancy. In our study, LVEF and EF from 3D STE also evidenced the uncompromised PRKAG2 LV systolic function.

To distinguish PRKAG2 syndrome from HCM, we combined HR and GLS for ROC curve analysis. For the best cut-off value (0.114), the sensitivity and the specificity were 69% and 100%, respectively. The progression of gene sequencing provides opportunities to identify patients with PRKAG2 syndrome from HCM patients. However, gene sequencing is limited by clinical availability and economic factors. Clinically, the suspicion is based on “red flags”, which traditionally include young age, unexplained LVH, bradycardia, ventricular preexcitation, and high-grade conduction disease [1]. LVH has been reported as the most common clinical phenotype of PRKAG2 syndrome [2]. Only one-third of PRKAG2 patients demonstrate preexcitation [10]. For PRKAG2 patients whose phenocopies mimic HCM, preserved GLS may also be used as “red flags”. The AUC for HR was 0.828 (95% CI, 0.646—1); the best cut-off value’s sensitivity and specificity (57 bpm) were 86.7% and 77.8%, respectively. When GLS was incorporated, the specificity increased to 100%, with the sensitivity decreasing to 69%. ECG examination has the potential for screening PRKAG2 syndrome. 3D STE can also be performed for more evidence of PRKAG2 syndrome.

### Limitations

The studied population is a sample of PRKAG2 syndrome with LVH. However, some PRKAG2 patients do not present LVH. The 3D STE characteristics of those patients are not investigated in this study. Another limitation is that the number of PRKAG patients enrolled was small and all the patients were from the same hospital.

## Conclusions

In conclusion, PRKAG2 patients present deteriorated LV diastolic function and preserved LV systolic function compared with healthy subjects. Bradycardia and preserved GLS have the potential to distinguish PRKAG2 syndrome from HCM with high specificity. Our study demonstrates that ECG and 3D STE are useful for identifying patients with PRKAG2 syndrome, which may help clinical decision-making.

## Supplementary Information

**Additional file 1: Figure S1.** Family pedigrees of PRKAG2 syndrome.

## Data Availability

The datasets used and/or analysed during the current study are available from the corresponding author on reasonable request.

## Abbreviations

LVH: Left ventricular hypertrophy
HCM: Hypertrophic cardiomyopathy
GLS: Global longitudinal strain
GCS: Global circumstantial strain
LVEF: Left ventricular ejection fraction
