# Supplementary figures and images for: Echocardiographic characteristics of PRKAG2 syndrome: a research using three-dimensional speckle tracking echocardiography compared with sarcomeric hypertrophic cardiomyopathy
Source: Cardiovasc Ultrasound. 2022 May 5;20:14. doi: 10.1186/s12947-022-00284-3 (PMC9069802; doi:10.1186/s12947-022-00284-3)

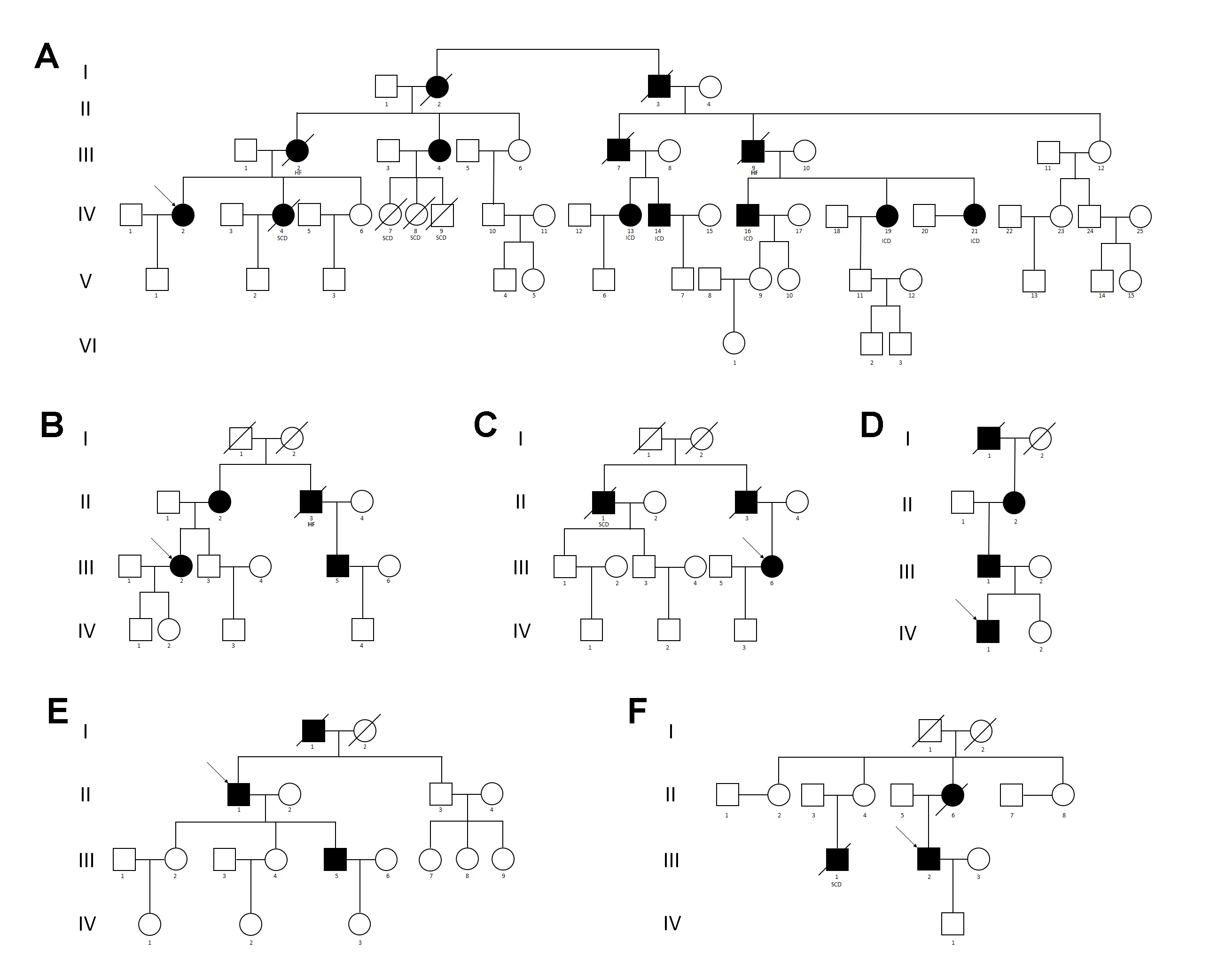

Supplement: Supplementary file 1 — Additional file 1: Figure S1. Family pedigrees of PRKAG2 syndrome. [file 12947_2022_284_MOESM1_ESM.jpg]
